# Evaluation of Biological Effect of *Triticum vulgare* Extract Compared With Hyaluronic Acid in In Vitro Tissue Models

**DOI:** 10.1155/adpp/8857215

**Published:** 2026-10-05

**Authors:** Carlo Oliviero, Alessandra Fusco, Enrica Giustino, Roberta Palladino, Eugenia Romano, Barbara Maglione, Antonella D’Agostino, Chiara Schiraldi

**Affiliations:** ^1^ Department of Experimental Medicine University of Campania “Luigi Vanvitelli” Naples Italy unina2.it; ^2^ Department of Life Sciences, Health and Health Professions Link Campus University Rome Italy; ^3^ Farmaceutici Damor S.p.A Naples Italy

**Keywords:** antioxidant activity, defensin-2, fibroblast, keratinocytes, tissue regeneration, *Triticum vulgare* extract, wound healing

## Abstract

Plant extracts and microbial‐derived molecules, particularly oligo‐ and polysaccharides, are known to promote tissue repair. *Triticum vulgare* extract (TVE), derived from germinated whole plants and utilized in medical devices, is rich in bioactive molecules comprised of oligo‐ and polysaccharides with diverse biological properties beneficial for wound repair. This study investigates the biochemical mechanisms underlying TVE’s proregenerative activity in vitro effects, comparing it with hyaluronic acid (HA), a gold standard in wound healing. Preliminary results showed that TVE has scavenging properties/antioxidant activity also at lower concentrations with respect to HA that has long been recognized for these properties (Galvez‐Martin et al.). In vitro assays evaluated cell proliferation, migration, extracellular matrix biomarkers, and responses to stress in 2D and 3D models. TVE significantly enhanced keratinocyte migration, reaching approximately 50% wound closure in less than 20 h, whereas the untreated control required about 30 h to achieve the same level of closure. Moreover, TVE performed better than HA at low concentrations, while both treatments improved HDF proliferation and ECM biomarker expression. More specifically, COL1 expression was upregulated by 2.3‐fold relative to the control, showing an effect comparable to that induced by HA (2.8‐fold). Conversely, ELS expression exhibited a 2.0‐fold increase in response to TVE and a 2.6‐fold increase following HA treatment compared with the control. In addition, the extract significantly increased HBD‐2 expression by 2‐fold relative to the control, a novel finding that may be relevant, given the role of this antimicrobial peptide in innate immunity, inflammation regulation, and wound repair. Under stressful conditions, including dehydration and exposure to reactive oxygen species, TVE showed protective effects comparable to HA in both HDF and HaCaT cells. 3D skin models, including those treated with LPS, confirmed the results of 2D assays, further validating TVE’s biological properties. Overall, these studies demonstrate that this extract exerts biological effects comparable to pharmaceutical HA in several in vitro skin models. These findings further support the potential application of TVE in wound management and skin regenerative approaches.

## 1. Introduction

In recent years, the rise in chronic skin lesions has become a growing concern for national health systems worldwide, accounting for approximately 2%–5% of total healthcare costs [1]. Among them, skin wound healing is a highly complex and dynamic process, involving the synchronized interaction of various cell types and chemical mediators over time. Triggered in response to tissue injury, the wound healing process aims to repair cellular and extracellular matrix (ECM) damage while restoring the integrity of the skin barrier. This process unfolds through four interrelated and overlapping phases: hemostasis, inflammation, proliferation, and remodeling [2]. Scientific literature reported various bioactive molecules are able to operate at different stages, facilitating and improving tissue repair. Among the bioactive compounds, a huge part derived from natural resources, and in particular plant extracts and natural polymers, are key components of commercial medical device commonly used in regenerative medicine [3–5].

In particular, plant extracts have shown beneficial effects in wound healing by promoting skin repair through enhanced bio‐adhesion, immunomodulation, cell–cell and cell–matrix interactions, and increased collagen synthesis [6]. Among plants, *Triticum vulgare* extract (TVE), an active component of Fitostimoline® developed by Damor Farmaceutici S.p.A., has been widely exploited in traditional medicine for its ability to accelerate tissue repair [7]. The extract is obtained from the whole germinated plant, according to a procedure described and patented by Sanguigno et al. and Riccio et al. [8, 9] and contains a mixture of oligo‐polysaccharide molecules and a small number of phytochemical compounds [10, 11]. TVE has been shown to improve in vitro proliferation and wound healing in different cellular models [8, 12]. In addition to wound closure, the extract improved by also regulating the expression of MMP‐2 and MMP‐9 over time and facilitating a structured and consistent matrix remodeling through the upregulation of key dermal biomarkers [12]. Other studies assessed that fibroblast migration in the presence of TVE is strongly associated with increased fibronectin synthesis, enhanced actin polymerization, and stimulated hyaluronan synthase 2 expression [13].

Further evidence suggested that *Triticum* extract may also attenuate the inflammatory response by reducing nitric oxide, IL‐6, PGE2, and TNF‐α levels in a rat microglia cell model [14], while it was found the intrinsic capacity of TVE to counteract oxidative stress derived from its scavenging activity against free radicals generally present in burns and injuries [15].

Similarly, hyaluronic acid (HA), a natural polymer and a key component of the ECM, over the years, played a crucial role in various stages of wound healing, adapting its function according to its molecular weight [16, 17].

In particular, HA with a molecular weight ranging from 100 to 300 kDa has shown a positive effect in accelerating wound closure by increasing the expression of ZO‐1 protein and P2X7 receptor [18]. Bearing in mind the state of the art, in this study, we further investigated the biological response of TVE also in comparison with pharmaceutical‐grade HA with a molecular weight of approximately 220 kDa. The latter was selected as the reference standard because HA within the low‐to‐intermediate molecular weight range is widely used in wound‐healing and tissue‐repair studies and has been reported to modulate cellular responses associated with these processes, including fibroblast migration, proliferation, ECM remodeling, and re‐epithelialization [15, 19, 20]. Specifically, the potential regenerative effect of TVE was evaluated and compared with those of HA treatments using different 2D cellular models (HaCaT and human dermal fibroblast [HDF] cells). In vitro stress conditions were also applied to assess the ability of these compounds to protect the skin from dehydration and oxidative stress, because common strategies in accelerating wound healing process may often correlate with an improved response to these further challenges besides wound itself [21]. Another approach to evaluate the efficacy of TVE in tissue regeneration, particularly relevant in the treatment of chronic lesions, was to assess its antimicrobial activity, which has not yet been explored. Human *β*‐defensin‐2 (HBD‐2) is a natural peptide produced by dermal cells, exhibiting activity against Gram‐positive and Gram‐negative bacteria as well as fungi, and was assessed in this context. Its expression in cultured epithelial cells is induced by proinflammatory cytokines, microorganisms, and other inflammatory factors. Beyond its antimicrobial function, the presence of HBD‐2 in the outer epidermal layers suggests a key role in innate immunity and in tissue repair processes [22–24].

A 3D skin system, designed to closely mimic the complexity of native skin, was employed to better understand the TVE biological response, evaluating specific biomarkers involved in skin regeneration and inflammation.

## 2. Materials and Methods

### 2.1. Materials

TVE was provided by Damor Farmaceutici S.p.A. (Naples, Italy), batch N°181127; it is a registered, patent‐protected pharmaceutical active ingredient manufactured by Farmaceutici Damor (Naples, Italy) under strict protocols and in agreement with the Good Manufacturing Practices (GMP) guidelines. A pharmaceutical‐grade sodium hyaluronate powder, with a declared molecular weight of 220 kDa, was kindly provided by Altergon Italia S.r.l. (Avellino, Italy). A molecular hydrodynamic characterization of the sample was performed as previously reported by Di Meo et al. [25]. Liquid TVE extract was diluted in the medium at a concentration of 0.1 mg/mL as previously reported [9]. HA powder was dissolved in the culture medium at the same concentration (0.1 mg/mL). Lipopolysaccharides (LPS; 5 × 10^6^ EU of VacciGrade™ lipopolysaccharide from E. coli A1:O111:B4) were purchased by InvivoGen (Toulouse, France) at a final concentration of 1 and 2 μg/mL in culture medium for HaCaT and 3D skin model, respectively, to simulate chronic wound.

#### 2.1.1. Cell Culture

Human keratinocytes (HaCaT) (supplied by “Zooprofilattico Institute”, Brescia, Italy) and HDF cell line immortalized with hTERT (HDF cells, BJ‐5ta, ATCC CRL‐4001), were cultured in Dulbecco’s modified Eagle medium (DMEM), supplemented with 10% fetal bovine serum (FBS), 1% penicillin–streptomycin, 1% gentamicin, and 1% amphotericin B. All the reagents were purchased by GIBCO (Life Technologies, Milan, Italy).

The Phenion® Full Thickness Skin Model, produced by Henkel (Düsseldorf, Germany, diameter 1.3 cm), was used for the 3D experiments. The pieces were appropriately cut and treated according to the manufacturer’s protocol, for real‐time PCR (RT‐PCR) and immunofluorescence (IF) assays [26].

### 2.2. Methods

#### 2.2.1. In Vitro Antioxidant Activity

To evaluate TVE and HA antioxidant activity, two in vitro colorimetric assays were performed: the 2,2‐diphenyl‐1‐picrylhydrazyl (DPPH) assay [27] and the 2,2′‐azino‐bis (3‐ethylbenzothiazoline‐6‐sulfonic acid) (ABTS) assay [28]. The DPPH assay was performed by incubating 1 mL of each sample, tested at 0.1, 0.5, 1, and 2 mg/mL, with 200 μL of ethanolic solution of 0.4 mM DPPH. After mixing, 2 mL of highly purified H_2_O (milliQ water) was added to each sample and vortexed, and the mixture was allowed to react for 30 min at room temperature in the dark. At the end of the incubation, the absorbance was measured spectrophotometrically at 517 nm. Ascorbic acid was used as standard. Results are reported as percentage of DPPH inhibition with respect to control (i.e., absorbance of the sample in the absence of any molecule), following the equation:

(1) Inhibition %=1−Abs517sampleAbs517control×100.

As for the ABTS assay, both molecules, tested at different concentrations, were allowed to react with ABTS^•+^ for 7 min in the dark, and the absorbance was measured at 734 nm. Ascorbic acid was used as a standard to obtain a calibration curve. Results are reported as a percentage of ABTS inhibition with respect to the control (i.e., absorbance of the sample in the absence of any molecule), following the equation:

(2) Inhibition %=1−Abs734sampleAbs734control×100.

#### 2.2.2. Dehydration Test

The dehydration tests were performed on HaCaT and HDF cell lines cultured in 24‐well plates at approximately 70% confluence to facilitate better observation of cell morphology. Cells were pretreated with 0.1 mg/mL TVE or HA for 2 h. After incubation, the treatments were removed (except for the positive control, CTR+, where DMEM with 10% FBS was maintained throughout the experiment), and the plate was left open in the incubator (37°C, 5% CO_2_, humidified air) for 30 min to simulate a dehydration‐stress condition, as described by d’Agostino et al. [26]. The dehydrated control (CTR‐) was processed following the same protocol without any treatment. Cell viability was assessed by the 3‐(4,5‐dimethylthiazol‐2‐yl)‐2,5‐diphenyl‐2H‐tetrazolium bromide (MTT) assay. Cell morphology was observed and recorded by an inverted optical microscope (Zeiss Observer Z.1 equipped with Zen Pro software) before and after dehydration to monitor cellular damage and stress.

#### 2.2.3. In Vitro Keratinocytes and HDF Scratch Test and Time‐Lapse Video Microscopy (TLVM)

Scratched HaCaT or HDF cell monolayers were used as a simplified model of human dermal tissue to evaluate the biological effects of TVE and HA formulations on wound closure. An injury approximately 0.5–0.9 mm wide was mechanically created with a sterile pipette tip (*Ø* = 0.1 mm) and washed with PBS to remove cellular debris. The scratched monolayers were treated with 0.1 mg/mL of TVE or HA in DMEM 1% FBS for all the experiment. The control was diluted at the same concentrations using PBS (Phosphate‐Buffered‐Saline, Gibco, Life Technologies, Milan, Italy). The in vitro wound healing assay was conducted using a TLVM (OKOLAB, Pozzuoli, Italy, equipped with Zen Pro software), as previously described [12]. Briefly, TLVM allows us to monitor cell migration in real time using a stage incubator to maintain in vitro cell culture conditions. Moreover, different multiwell plates can be accommodated in the stage incubator, allowing control and sample replicates to be analyzed simultaneously within a single experiment and thereby providing statistically significant results [29]. Each experimental condition was tested in biological triplicate and was independently repeated to ensure reproducibility. For image analysis, multiple fields per well were acquired and analyzed to obtain representative quantitative data of wound closure over time. For each acquired image, the scratched area was measured at time zero (A (*t*_0_)) and every hour (A (t)). The residual scratched area (%) over time, normalized to A (*t*_0_) was calculated as follows:

(3) AtAt0×100.

#### 2.2.4. Proliferation Assay

HDF cell proliferation was monitored at 24, 48, and 72 h by using the Cell Counting Kit‐8 (CCK‐8) (Dojindo Laboratories, Japan), a noncytotoxic colorimetric assay for the quantification of cell viability over time. Briefly, 3 × 10^4^ cells/well were seeded in a 24‐well plate for 24 h and then treated with 0.1 mg/mL of either TVE or HA. After incubation, the medium containing the formulations was removed, and the CCK‐8 reagent was added according to the manufacturer’s instructions. The experiment was performed in triplicate.

#### 2.2.5. RNA Isolation and Quantitative Gene Expression by qRT‐PCR on HaCaT and HDF Cells

Wound healing biomarker gene expressions were evaluated 24 h post‐scratch in HaCaT and HDF cells. Cells were lysed using TRIzol® (Invitrogen, Milan, Italy), and the RNA was extracted, quantified with a NanoDrop spectrophotometer (Celbio, Milan, Italy), and reverse‐transcribed to obtain cDNA by using the SensiFAST cDNA System Kit (Bioline). The cDNA was combined with iQ™ SYBR® Green Supermix (Bio‐Rad Laboratories Srl). qRT‐PCR was performed by using specifically designed primers for *COL1A1, COL3A1*, *ELS,* and *HBD-2* (Beacon Designer™) (Table 1).

**TABLE 1:** Primer sequences used for qRT‐PCR.

Gene	Forward primer	Reverse primer	T (°C)
COL1A1	5′‐CCAGAAGAACTGGTACATCA‐3′	5′‐CCGCCATACTCGAACTGGAA‐3′	57
COL3A1	5′‐GTCCCCAAGGTGTCAAAG‐3′	5′‐GGGGGTCCTGGGTTACCATTA‐3′	58
ELS	5′‐AGGTGTATACCCAGGTGGCGTGCT‐3′	5′‐CAACCCCTGTCCCTGTTGGGTAACTG‐3′	57
GAPDH	5′‐TTCCACGGCACAGTCAA‐3′	5′‐GCAGGTCAGGTCCACAA‐3′	57
HBD‐2	5′‐GGATCCATGGGTATAGGCGATCCTGTTA‐3′	5′‐AAGCTTCTCTGATGAGGGAGCCCTTTCT‐3′	63

All samples were analyzed in triplicate. Gene expression was normalized to *GAPDH* and compared to controls. For 3D models, after 72 h, the samples were previously disrupted using a homogenizer to facilitate RNA extraction.

#### 2.2.6. IF Analyses

HDF cells (5 × 10^3^ cells/well) were seeded and cultured on chamber slides (BD Falcon, Franklin Lakes, NJ, USA) under control conditions and in the presence of TVE and HA for 24 h to assess the expression of COL1A1, COL3A1, and ELS proteins via IF assay. After incubation, the supernatants were removed, and the samples were washed twice with PBS, fixed with 4% w/v paraformaldehyde, and permeabilized with 0.2% v/v Triton X‐100 in PBS. Primary antibodies against COL1A1, COL3A1, and ELS (Abcam, Cambridge, UK; each diluted 1:100) were applied and incubated overnight at 4°C. Subsequently, samples were incubated for 1 h at room temperature with FITC‐conjugated goat anti‐rabbit and goat anti‐mouse secondary antibody (Thermo Fisher Scientific, Waltham, MA, USA; each diluted 1:200). Following this, the samples were stained with Phalloidin for 40 min at room temperature and mounted with an aqueous medium containing DAPI. Images were acquired using a Nikon fluorescence microscope and analyzed using Nikon Nis‐Elements software (Version D.4.00). For the 3D model, IF protocol reported in d’Agostino et al. was accomplished [26].

IF images were analyzed using Fiji/ImageJ software (National Institutes of Health, Bethesda, MD, USA). Images were acquired under identical microscope settings and analyzed in 8 bit format. Fluorescence images were converted from RGB format to grayscale to allow intensity measurements. For each image, the mean gray value corresponding to the mean fluorescence intensity (MFI) was measured over the entire image area. Background fluorescence was determined from a region devoid of specific staining within the same image. Background corrected fluorescence intensity was calculated according to the following equation:

(4) Corrected MFI=Mean Gray Value Image‐Mean Gray Value Background.

where Mean Gray Value Image represents the average fluorescence intensity measured across the entire image and Mean Gray Value Background represents the average intensity measured in a signal free area.

Corrected fluorescence intensities were expressed as arbitrary units (a.u.). Measurements obtained from independent images were averaged and reported as mean ± standard deviation (SD).

#### 2.2.7. HBD‐2 Quantification by Enzyme‐Linked Immunosorbent Assay (ELISA)

Supernatants of scratched HaCaT cells (treated and untreated) and supernatants of 3D skin previously treated with LPS and after being scratched were collected after 48 and 72 h, respectively, and analyzed using ELISA for quantification of HBD2 production (Elabscience) according to the manufacturer’s instructions. The assay was carried out in triplicate.

#### 2.2.8. Reparative Effects Against H_2_O_2_‐Induced Oxidative Stress

TVE and HA were evaluated for their ability to repair the effect of H_2_O_2_‐induced oxidative stress in HaCaT and HDF cell lines. Cells were cultured in a 24‐well plate until they reached approximately 70% confluence to allow for optimal observation of cell morphology. After the removal of the culture medium, cells were washed with PBS and then exposed to 50 μM H_2_O_2_ (Sigma‐Aldrich, Italy) diluted in medium to induce oxidative stress for 30 min. The choice of this concentration, 50 μM H_2_O_2_, represents a well‐established experimental condition for modeling oxidative stress in keratinocytes while preserving sufficient cell viability for functional assays and it is already used also in our previous research [30]. Morphological changes due to oxidative damage were assessed before and 24 h after treatment using TLVM.

Following the oxidative stress induction, H_2_O_2_ solution was removed, cells were washed with PBS, and cells were subsequently treated with TVE and HA for 24 h. After the treatment incubation, cell viability was assessed using the MTT assay.

#### 2.2.9. IL‐6 Expression by ELISA

Supernatants from scratched HaCaT cells (24h) and scratched 3D skin models (72 h), previously stimulated with LPS, were collected to assess IL‐6 production via an ELISA, performed according to the manufacturer’s protocol (Boster Antibody and ELISA Experts, Tema Ricerca, Padova, Italy). Following collection, the supernatants were centrifuged at 3000 rpm for 10 min at 4°C, and aliquots were transferred, in triplicate, to a microtiter plate for incubation with a biotinylated polyclonal antibody specific for IL‐6. Optical density was measured at 450 nm using a microplate reader (Bio‐Rad Laboratories S.r.l., Segrate, Italy). IL‐6 concentrations were calculated using a standard calibration curve, according to the manufacturer’s instructions.

#### 2.2.10. Statistical Analysis

Each experiment was performed at least in triplicate, and results are reported as the mean value and SD. Data were statistically evaluated using an ANOVA test, with Tukey post‐hoc correction for multiple comparison. The level of significance was marked in figures, and *p* values lower than 0.05 indicated statistical differences.

## 3. Results

### 3.1. In Vitro Antioxidant Activity

As the emergence of oxidative stress is often related to the onset of related pathologies, the potential antioxidant activity of TVE and HA was assessed. In particular, different concentrations of either TVE or HA (0.1, 0.5, 1, and 2 mg/mL) were tested for their ability to scavenge DPPH and ABTS radicals, compared with the gold standard ascorbic acid, as reported in Material and Methods section. Interestingly, as shown in Figure 1, where the percentage of inhibition is reported for both samples as a function of the concentration analyzed, both TVE and HA exerted an antioxidant activity, even if at different extent. At the lowest concentration tested, 0.1% mg/mL, TVE showed a slight but increasing inhibitory activity with respect to HA. Indeed, TVE showed a 60% inhibition of DPPH radical (Figure 1(a), light blue bars), comparable among the tested concentrations. On the other hand, the inhibitory activity against ABTS showed a dose‐dependent trend, up to almost 90% inhibition at 2 mg/mL (Figure 1(b), light blue bars). Different was the case of HA, in which the inhibitory activity measured was significantly lower than the TVE one, around 35% for DPPH (Figure 1(a), blue bars) and 25% for ABTS (Figure 1(b), blue bars).

**FIGURE 1:**
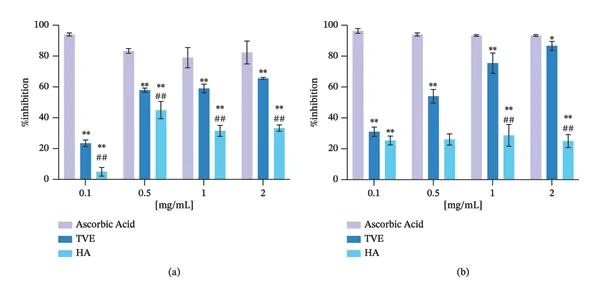
Antioxidant activity evaluation by in vitro DPPH and ABTS assay**.** Percentage of inhibition of DPPH (a) and ABTS (b) radicals for ascorbic acid (purple bars), TVE (blue bars), and HA (light blue bars) tested at 0.1, 0.5, 1, and 2 mg/mL. ^∗^ *p* < 0.05, ^∗∗^ *p* < 0.01, with respect to ascorbic acid. ^##^ *p* < 0.01 with respect to TVE.

### 3.2. Cellular model: HaCaT

#### 3.2.1. Effect of TVE and HA in Wound Healing

TVE and HA treatments were added to scratched HaCaT monolayers to assess their ability to promote tissue regeneration. The panel (Figure 2(a)) showed a qualitative and direct representation of the repair process. As shown in Figure 2(b), TVE demonstrated increased effectiveness in accelerating wound closure compared to the control (CTR) cells. Among the treatments, TVE achieved approximately 20% and 50% wound closure significantly earlier than HA and CTR, respectively (Table 2). The wound was nearly completely repaired within 40 h following TVE treatment, whereas the other groups showed a persistent scratch with only 60%–70% closure.

**FIGURE 2:**
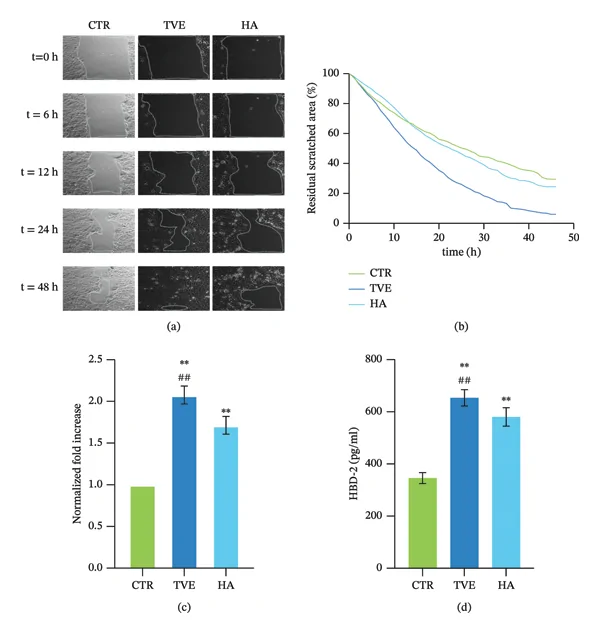
Wound healing assay. (a) Representative pictures of HaCaT monolayers after the scratch and over the time course of the experiment. (b) Quantitative analysis of wound closure over time. HBD‐2. (c) qRT‐PCR relative to *HBD-2* expression in the presence of different treatments at 24 h. (d) ELISA relative to HBD‐2 protein expression (pg/mL) in the CTR and in the presence of different treatments at 48h. ^∗∗^ *p* < 0.01 compared to CTR, ^##^ *p* < 0.01 compared to HA.

**TABLE 2:** Time to achieve 20%, 50%, and 70% wound closure in the CTR and in the presence of TVE and HA.

Sample	Time to reach x% of scratch repair		
	20%	50%	70%
CTR	7.5 ± 2.4	24.3 ± 7.3	37.1 ± 6.1
TVE	6.2 ± 1.4^∗^	14.0 ± 2.4^#^	25.1 ± 6.9^∗^, ^#^
HA	9.0 ± 1.3	23.3 ± 5.5	34.5 ± 6.0

^∗^ *p* < 0.05 compared to CTR.

^#^ *p* < 0.05 compared to HA.

#### 3.2.2. HBD‐2 Gene and Protein Expression by ELISA

For the first time, the effect of TVE on the expression of *HBD-2* was detected. qRT‐PCR results showed a slightly higher expression of *HBD-2* in TVE‐treated cells compared to CTR after 24h of incubation which a similar effect observed for HA (Figure 2(c)). The expression of HBD‐2 protein after 48h increased significantly in the presence of treatments with respect to the CTR, especially for TVE (Figure 2(d)).

#### 3.2.3. IL‐6 via ELISA

A slight but significant increase in IL‐6 was found in the presence of LPS, and both TVE and HA reduced the cytokines back to the control level (Figure 3).

**FIGURE 3:**
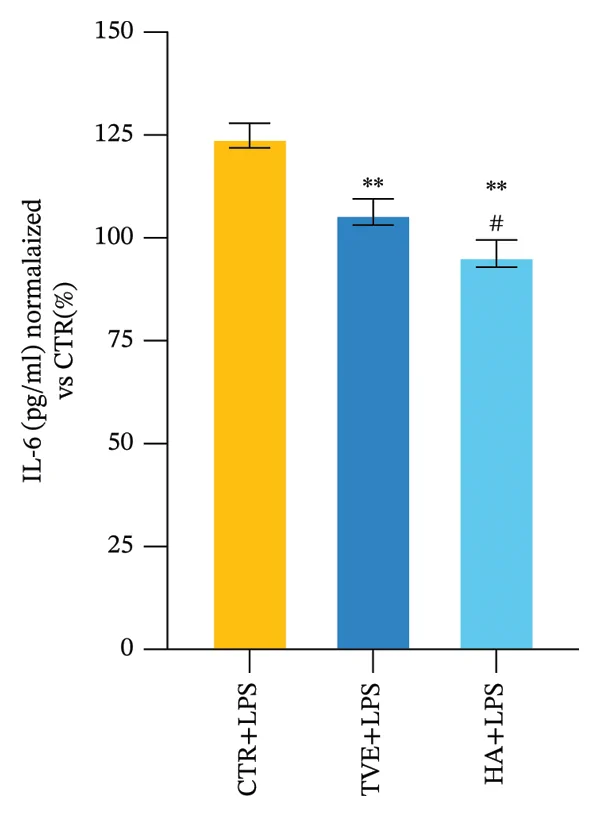
ELISA in scratched HaCaT pretreated with LPS in the CTR and in the presence of treatments TVE and HA. ^∗∗^ *p* < 0.01 compared to CTR + LPS, ^#^ *p* < 0.05 compared to TVE.

### 3.3. Cellular Model: Immortalized HDF

#### 3.3.1. Proliferation and IF

To assess the ability of the extract to enhance cell proliferation compared to the control, fibroblasts were treated with TVE and HA for 24, 48, and 72 h, and cell growth was monitored using CCK‐8. As shown in Figure 4(a), both TVE and HA significantly promoted cell proliferation at 48 and 72 h, with statistical significance only at 48 h.

**FIGURE 4:**
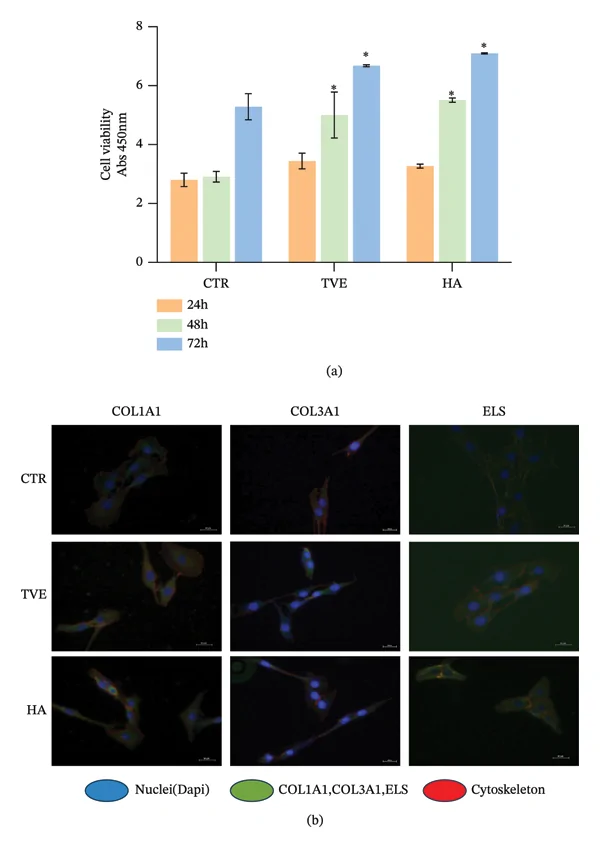
Proliferation and Immunofluorescence. (a) CCK‐8 assay, Proliferation of HDF‐Htert in the control and in the presence of TVE and HA at 24, 48, and 72 h. (b) Immunofluorescence of COL1A1, COL3A1, and ELS staining after 24 h of the treatments. Magnification: 400×. ^∗^Indicates *p* < 0.05 compared to CTR.

Regarding protein expression, IF analysis revealed higher levels of COL1A1, COL3A1, and ELS in treated cells compared to the control group (Figure 4(b)). To further validate these observations, fluorescence intensity was quantified using Fiji/ImageJ. Quantitative analysis confirmed an increase in COL1A1 expression in both treatment groups, with corrected MFI values increasing from 0.81 ± 0.22 a.u. in the CTR group to 1.86 ± 0.65 a.u. and 2.31 ± 0.15 a.u. in the TVE and HA groups, respectively. Similarly, ELS fluorescence intensity increased from 0.70 ± 0.34 a.u. in CTR cells to 1.37 ± 0.77 a.u. and 1.81 ± 0.16 a.u. following TVE and HA treatment, respectively. COL3A1 expression was also enhanced in treated cells, with the highest fluorescence intensity observed in the TVE group (1.56 ± 0.38 a.u.), compared with HA‐treated (0.77 ± 0.07 a.u.) and control cells (0.48 ± 0.01 a.u.). All data were reported in Table 3. Overall, these findings corroborate the qualitative IF observations and suggest that both treatments promote ECM protein production, with TVE showing a particularly pronounced effect on COL3A1 expression.

**TABLE 3:** Quantitative analysis of extracellular matrix protein expression in 2D fibroblast cultures.

Marker	Sample		
	CTR (a.u.)	TVE (a.u)	HA (a.u)
COL1A1	0.81 ± 0.22	1.86 ± 0.65	2.31 ± 0.15
ELS	0.70 ± 0.34	1.37 ± 0.77	1.81 ± 0.16
COL3A1	0.48 ± 0.01	1.56 ± 0.38	0.77 ± 0.07

#### 3.3.2. Wound Healing Assays

Representative panels of the wound healing experiment (supporting S1) confirmed that no appreciable difference was found in the control and in the presence of treatments [25].

#### 3.3.3. ECM Gene Expression

The effect of TVE was evaluated by evaluating the expression levels of *COL1A1*, *ELS,* and *COL3A1* after 24 h of treatment (Figure 5). All the treatments increased the normal expression of these ECM genes. qRT‐PCR results showed slightly better expression of *ELS* by TVE with respect to HA, but the *COL1A1* and *COL3A1* expression by HA is better with respect to TVE. For TVE treatments, the fold increase was 1.5, 3, and 3 with respect to the CTR, while for HA, *COL1A1*, *ELS,* and *COL3A1* showed an upregulation of 2.8, 2.5, and 5.2, respectively, compared with untreated cells.

**FIGURE 5:**
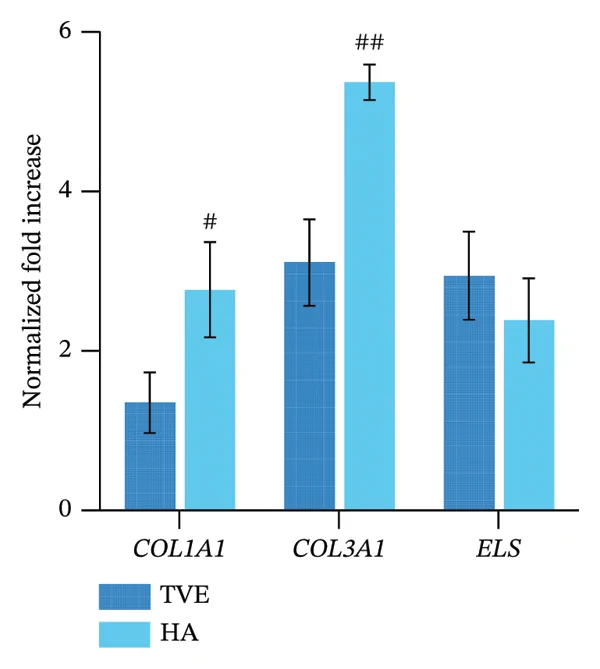
qRT‐PCR of scratched HDF relative to *COL1A1*, *COL3A1*, and *ELS* expression in the presence of different treatments at 24h. ^#^ *p* < 0.05 and ^##^ *p* < 0.01 compared to TVE.

#### 3.3.4. Analysis of Protective Efficacy of TVE Against Dehydration

The dehydration test in HaCaT and HDF cells, in terms of cell viability, is shown in Figure 6(a). Data normalized to the CTR + group confirmed the protective effects of both TVE and HA against dehydration‐induced stress. In HaCaT cells, viability increased from 10.73 ± 1.09% in the CTR− group to 25.08 ± 7.61% and 25.00 ± 8.02% following TVE and HA treatment, respectively. Similarly, in HDF cells, viability increased from 10.53 ± 4.44% in the CTR− group to 21.98 ± 2.82% and 22.49 ± 4.29% after TVE and HA treatment, respectively. These correspond to approximately 2.3‐fold increases in viability compared with the dehydrated control. Both treatments showed statistically significant differences compared to the CTR− group, while no significant differences were observed between TVE and HA.

**FIGURE 6:**
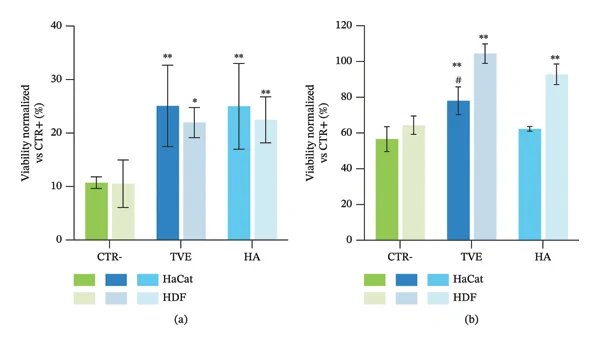
Dehydration test (a) Viability assay by MTT test. Reparative effects in HaCaT and HDF (b). Cell viability by MTT assay. ^∗^ *p* < 0.05, ^∗∗^ *p* < 0.01 compared to CTR‐ and ^#^ *p* < 0.05 compared to HA.

#### 3.3.5. Reparative Effects Against H_2_O_2_‐Induced Oxidative Stress

The reparative effect of treatments following stress induction is shown in Figure 6(b). In HaCaT cells, cell viability in the control group decreased to approximately 50% after stress. Treatment with TVE led to a notable recovery, with cell viability reaching around 80%. In contrast, HA treatment showed a less pronounced effect and did not produce a statistically significant improvement compared to the control. In HDF cells, cell viability in the control group decreased to approximately 60% after stress. Treatment with TVE and HA led to a notable recovery, with cell viability reaching around 100% and 90%, respectively.

### 3.4. Full‐Thickness 3D Skin Model

#### 3.4.1. Gene and Protein Expression of Biorevitalizing and Inflammation Biomarkers

The expression of *COL1A1*, *COL3A1,* and *ELS* in 3D models treated with LPS and successively scratched and incubated for 72 h is reported in Figure 7. All the genes were slightly upregulated with respect to the control. No statistical differences were found between the treatments.

**FIGURE 7:**
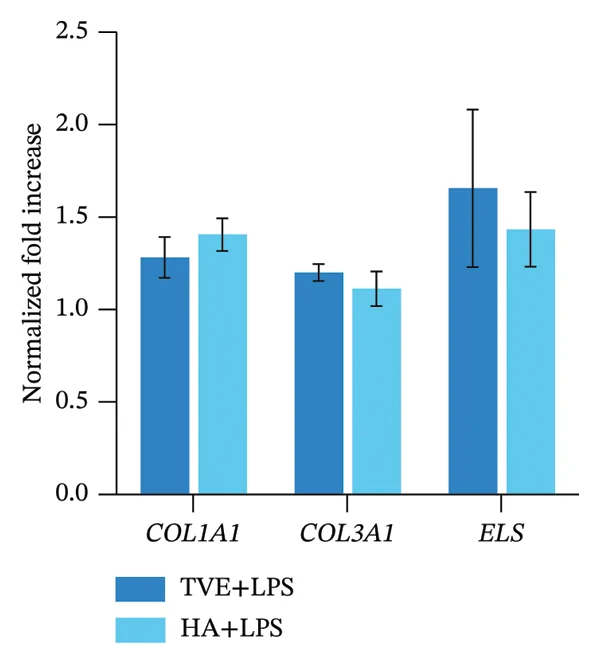
qRT‐PCR of 3D skin treated with LPS and after scratching relative to *COL1A1*, *COL3A1,* and *ELS* expression in the presence of different treatments at 72 h.

IF images (Figure 8) further supported the transcriptional findings and provided quantitative evidence of ECM protein deposition. Fluorescence intensity analysis revealed increased COL1A1 expression in both treatment groups compared with the control, with corrected MFI values of 1.47 ± 0.27 a.u. for TVE and 1.53 ± 0.30 a.u. for HA, compared with 0.89 ± 0.21 a.u. in the CTR group. Consistent with the qualitative observations, ELS staining was more pronounced in treated constructs, particularly following TVE stimulation, reaching 3.27 ± 1.05 a.u., compared with 2.40 ± 0.68 a.u. for HA and 1.71 ± 0.60 a.u. in the control group.

**FIGURE 8:**
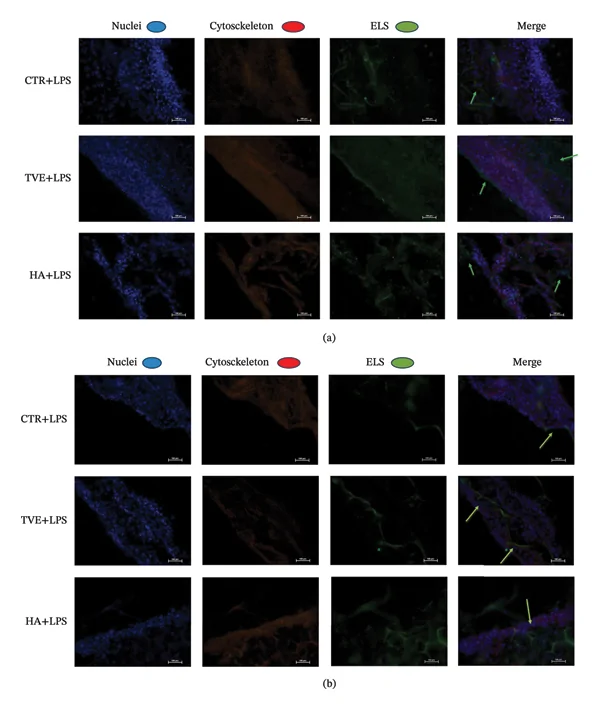
Immunofluorescence of ELS (a) and COL1A1 (b) on 3D skin treated with LPS and after scratching after 72 h of the treatments. Magnification 400X.

All data are reported in Table 4. Overall, the fluorescence quantification confirmed the trends observed in the representative images, indicating enhanced ECM deposition in response to both treatments, with TVE promoting elastin production and HA showing a slightly greater effect on COL1A1 expression.

**TABLE 4:** Quantitative analysis of extracellular matrix protein expression in 3D models.

Marker	Sample		
	CTR (a.u.)	TVE (a.u)	HA (a.u)
COL1A1	0.89 ± 0.21	1.47 ± 0.27	1.53 ± 0.30
ELS	1.71 ± 0.60	3.27 ± 1.05	2.40 ± 0.68

#### 3.4.2. Gene and Protein Expression of HBD‐2, IL‐6

IL‐6 produced by the 3D skin following treatment with LPS and injury is reported in Figure 9. Even if the increase is not marked, IL‐6 reduction was significant upon treatments with TVE and HA similarly (*p* < 0.05 with respect to CTR + LPS). The HBD‐2 expression was upregulated in the presence of LPS with respect to untreated CTR, whereas decreased following the addition of HA and especially of TVE (Figure 10). The results of ELISA confirmed these data. Moreover, TVE significantly decreased HBD‐2 protein levels compared with HA treatment (*p* < 0.05).

**FIGURE 9:**
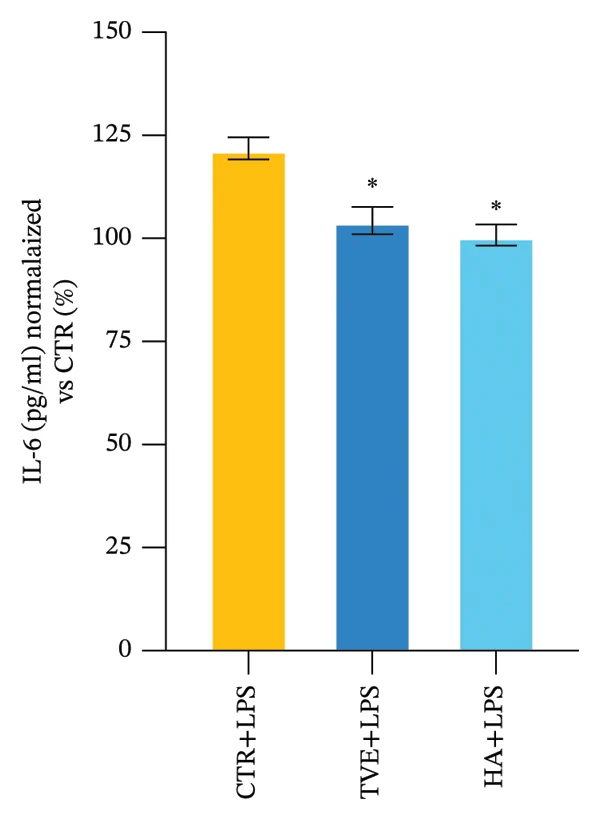
IL‐6 ELISA in scratched 3D model pretreated with LPS and then with TVE and HA. ^∗^ *p* < 0.05 compared to CTR + LPS.

**FIGURE 10:**
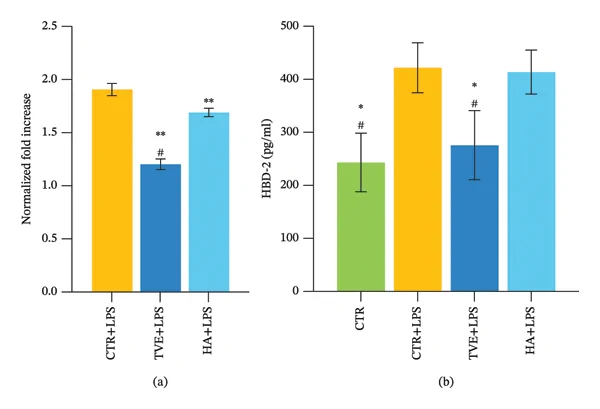
(a) qRT‐PCR and (b) ELISA of 3D skin treated with LPS and after scratched relative to *HBD-2* expressions in the presence of different treatments at 72h. ^∗^ *p* < 0.05, ^∗∗^ *p* < 0.01 compared to CTR + LPS. ^#^ *p* < 0.05 compared to HA.

## 4. Discussion

The present study provides a comprehensive characterization of the biological activity of TVE in a dermal in vitro model by directly comparing its effects with those of HA. In fact, hyaluronan is widely recognized for its role in skin repair and wound healing [30]. The ability of TVE to promote keratinocyte migration has been previously reported [11]; however, the present findings confirm this activity and further demonstrate that its beneficial effects extend beyond re‐epithelialization. Although TVE did not significantly enhance fibroblast migration, it was associated with the upregulation of ECM‐related genes also in this cell model, suggesting a prominent role in tissue remodeling. Moreover, by evaluating TVE across multiple in vitro models, including dehydration, oxidative stress, inflammatory wound‐like conditions and a 3D skin construct, this study expands the current understanding of its multifunctional biological profile and supports its potential relevance in different phases of dermal repair.

In detail, our data confirmed the efficacy of TVE in prompting cell migration, as reported elsewhere, and surprisingly, this effect was superior with respect to HA treatments at the same concentration. It has been reported that the efficacy of TVE in wound healing might be attributed solely to oligo‐ and polysaccharide fractions [8]. However, mass spectrometry analyses, recently reported by Monti and collaborators, found low‐weight phytochemical constituents, in particular ferulic acid, luteolin, and allantoin known to exert various beneficial effects [10]. Therefore, the increase in the cellular repair rate found in our experiments might be partly ascribed to these compounds that are reported to have effects in the different stages of wound repair. For instance, Kim et al. reported that ferulic acid prompted wound closure, inhibiting *β*‐catenin signaling, whose nuclear accumulation impairs keratinocyte motility [32]. In this respect, further studies are needed to better clarify the actual efficacy of these phytochemical compounds, particularly ferulic acid that is the most abundant, alone or in combination with the TVE oligosaccharides fraction [11].

In HDF scratched monolayers, TVE treatments did not show significant differences in repair compared to CTR and HA. These results are consistent with the recent literature, which reported no difference between CTR and HA (∼220 kDa) treatment in fibroblast migration [25]. Notably, the improvement in HDF scratch repair was mainly associated with increased expression of ECM‐related markers in TVE‐treated cells compared with the control, suggesting that repair is predominantly driven by matrix remodeling. In particular, this research work proved positive modulation of COL1A1 and ELS in proliferation assays, indicating the potential beneficial effect in agreement with previous evidence reported by Tito and co‐authors [13].

TVE was investigated under in vitro stress conditions in two dermal cell models, addressing previously unexplored aspects of its activity. In fact, TVE reduced dehydration‐associated changes to an extent comparable to that of HA, considered among the most effective biopolymers in this respect [26, 31]. Besides, both TVE and HA showed antioxidant activity already at low concentrations (0.1–0.5 mg/mL) with slightly higher performance for TVE [33]. These findings further support the possibility that TVE influences multiple cellular responses relevant to skin homeostasis.

However, the dehydration model used here represents an acute in vitro dehydration challenge and does not fully reproduce the complexity of physiological skin injury or wound healing in vivo, where barrier function, vascular supply, and immune responses, besides ECM remodeling, contribute substantially to tissue repair. Therefore, the dehydration results should be interpreted as evidence of protective effects under simplified stress conditions rather than as a direct demonstration of regenerative efficacy in vivo.

To further characterize the effects of TVE on dermal repair, we employed a scratch injury combined with LPS stimulation to simulate chronic wounds in vitro. Although this model does not fully recapitulate the complexity of chronic wounds, such as immune cell infiltration, vascular dysfunction, and biofilm‐associated infections, it reproduces one of the key features regarding the persistent inflammatory microenvironment. Therefore, it represents a useful and widely adopted experimental approach for the preliminary evaluation of wound healing therapies under proinflammatory conditions. Hence, TVE treatments enhanced ECM biomarkers expression compared to control, even in the presence of LPS, supporting its role in skin inflammation‐related disorders. In addition, IL‐6, among the most investigated biomarkers of chronic wound, was similarly reduced by TVE and HA‐treated samples in both models. The observed effect might be due to the combined action of the oligosaccharide fraction and luteolin since this flavonoid is recognized for its ability to reduce IL‐6 levels [34].

Further results showed that both TVE and HA addition prompted HBD‐2 expression, confirming their involvement in modulating innate immunity [23] [35–37].

Interesting results were found in a 3D full‐thickness skin model where the expression of HBD‐2 activated by LPS was, instead, downregulated in the presence of TVE. In this experimental setup, LPS is resembling wound infection, which leads to tissue alert and production of beta defensin‐2, while TVE counteracted the inflammatory state, and thus HBD‐2 expression was reduced [37–39].

Considering all the diverse experimental models reported here and the results obtained, it can be argued that the properties of the TVE include antioxidant, anti‐inflammatory, and innate immunity activities, further supporting the beneficial role of TVE in wound healing. Furthermore, taking advantage of a 3D full‐thickness skin model, a more complex in vitro system, better resembling in vivo conditions, was investigated. The results obtained improved knowledge on the diverse beneficial activities without implementing animal studies, in agreement with the most recent guidelines with a marked outlook on sustainability.

## 5. Conclusions

Overall, this work extends the current knowledge on TVE activity by demonstrating that its biological function is not limited to promoting wound closure but involves multiple complementary mechanisms relevant to tissue repair, including modulation of ECM remodeling, inflammatory responses, oxidative stress, and innate immune‐related pathways. The combined evaluation in conventional monolayer cultures, inflammatory conditions and advanced 3D skin constructs provides a broader and physiologically relevant characterization of TVE and supports its potential as a multifunctional ingredient for wound‐healing formulations. These findings strengthen the rationale for further translational studies aimed at validating its clinical relevance in skin repair applications.

## Author Contributions

C.S.: conceptualization, methodology, review and editing, funding acquisition, project administration, and supervision; A.D.: conceptualization, methodology, original draft preparation, writing, review and editing, formal analysis, investigation, and data curation; C.O.: investigation, writing, original draft preparation, editing, formal analysis, and data curation; A.F. and E.G.: methodology, formal analysis, and data curation. R.P., E.R., and B.M. are employees of DAMOR Farmaceutici Italia, and handled review and supervision.

## Funding

This research was funded by Progetto PON03PE_00060_3 CUP Ricerca B68C14000010005; the Grant for PhD studies of M.D. was partially financed by Damor Farmaceutici SPA within the program DM 117/2023 PNRR (M4C2—“Dalla Ricerca all′Impresa”). This research was also funded by BioTekNet SCpA.

Open access publishing facilitated by Universita degli Studi della Campania Luigi Vanvitelli, as part of the Wiley ‐ CRUI‐CARE agreement.

## Conflicts of Interest

R.P., E.R., and B.M are enrolled in Damor Farmaceutici; they were involved in the preparation of the extract, in the data curation, and in editing of the manuscript; however, they were not involved in the experimental setup of biological assays that were run at the Department of Experimental Medicine.

All the authors from the University of Campania “Luigi Vanvitelli” declare no conflict of interest.

## Supporting Information

Additional supporting information can be found online in the Supporting Information section.

## Supporting information


**Supporting Information 1** Supporting information 1 shows representative panels of the HDF‐hTERT wound healing assay, providing qualitative evidence that the treatments did not markedly improve cell migration compared with the untreated control.

**Supporting Information 2** Supporting information 2 shows representative images of the dehydration test performed on the different formulations. The images provide a visual overview of the samples analyzed, complementing the quantitative data reported in the corresponding main figure.

## Data Availability

Data will be made available on request from the authors.
