# Supplementary material for: Evaluation of Biological Effect of Triticum vulgare Extract Compared With Hyaluronic Acid in In Vitro Tissue Models
Source: Adv Pharmacol Pharm Sci. 2026 Oct 5;2026:8857215. doi: 10.1155/adpp/8857215 (PMC13639547; doi:10.1155/adpp/8857215)
Supplement: Supplementary file 1 [file ADPP-2026-8857215-s001.pdf]

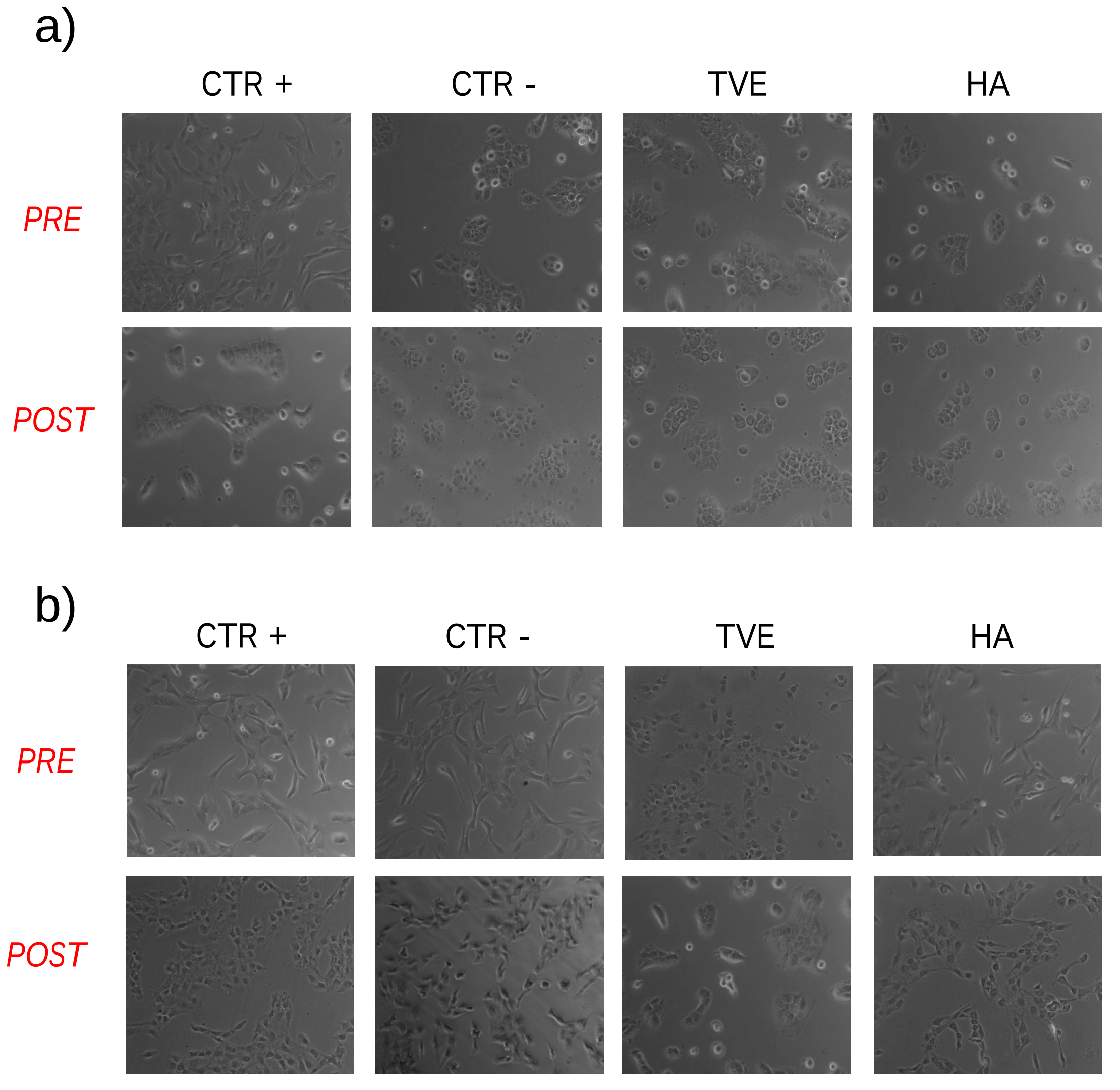
a)
CTR +
CTR -
TVE
HA
PRE
POST
b)
CTR +
CTR -
TVE
HA
PRE
POST
